# Clinical added value of MRI to CT in patients scheduled for local therapy of colorectal liver metastases (CAMINO): study protocol for an international multicentre prospective diagnostic accuracy study

**DOI:** 10.1186/s12885-021-08833-1

**Published:** 2021-10-18

**Authors:** B. Görgec, I. Hansen, G. Kemmerich, T. Syversveen, M. Abu Hilal, E. J. T. Belt, R. H. C. Bisschops, T. L. Bollen, K. Bosscha, M. C. Burgmans, V. Cappendijk, M. T. De Boer, M. D’Hondt, B. Edwin, H. Gielkens, D. J. Grünhagen, P. Gillardin, P. D. Gobardhan, H. H. Hartgrink, K. Horsthuis, N. F. M. Kok, P. A. M. Kint, J. W. H. Kruimer, W. K. G. Leclercq, D. J. Lips, B. Lutin, M. Maas, H. A. Marsman, M. Morone, J. P. Pennings, J. Peringa, W. W. Te Riele, M. Vermaas, D. Wicherts, F. E. J. A. Willemssen, B. M. Zonderhuis, P. M. M. Bossuyt, R. J. Swijnenburg, Å. A. Fretland, C. Verhoef, M. G. Besselink, J. Stoker, for the CAMINO Study Group, C. Bnà, C. De Meyere, W. A. Draaisma, M. F. Gerhards, F. Imani, K. F. D. Kuhlmann, M. S. L. Liem, Y. Meyer, J. S. D. Mieog, G. P. Serafino, H. C. Van Beek, J. A. B. Van der Hoeven, C. J. Veeken

**Affiliations:** 1 Department of Surgery, Amsterdam UMC, University of Amsterdam, Cancer Center Amsterdam, Amsterdam, The Netherlands; 2 Department of Radiology and Nuclear Medicine, Amsterdam UMC, University of Amsterdam, Cancer Center Amsterdam, Amsterdam, The Netherlands; 3 Department of Hepato-Pancreato-Biliary Surgery, Oslo University Hospital, Oslo, Norway; 4 The Intervention Centre, Oslo University Hospital – Rikshospitalet, Oslo, Norway; 5 Department of Radiology and Nuclear Medicine, Oslo University Hospital, Oslo, Norway; 6 Department of Surgery, Poliambulanza Foundation Hospital, Brescia, Italy; 7 Department of Surgery, Albert Schweitzer Hospital, Dordrecht, The Netherlands; 8 Department of Radiology, Albert Schweitzer Hospital, Dordrecht, The Netherlands; 9 Department of Radiology, St. Antonius Hospital, Nieuwegein, The Netherlands; 10 Department of Surgery, Jeroen Bosch Hospital, ‘s-Hertogenbosch, The Netherlands; 11 Department of Radiology, Leiden University Medical Center, Leiden, The Netherlands; 12 Department of Radiology, Jeroen Bosch Hospital, ’s-Hertogenbosch, The Netherlands; 13 Department of Surgery, University Medical Centre Groningen, Groningen, The Netherlands; 14 Department of Digestive and Hepatobiliary/Pancreatic Surgery, Groeninge Hospital, Kortrijk, Belgium; 15 Department of Radiology, Medical Spectrum Twente, Enschede, The Netherlands; 16 Department of Surgical Oncology, Erasmus Medical Center, Rotterdam, The Netherlands; 17 Erasmus MC Cancer Institute, Erasmus Medical Center, Rotterdam, The Netherlands; 18 Department of Radiology, Hospital Oost-Limburg, Genk, Belgium; 19 Department of Surgery, Amphia Hospital, Breda, The Netherlands; 20 Department of Surgery, Leiden University Medical Centre, Leiden, The Netherlands; 21 Department of Radiology and Nuclear Medicine, Amsterdam UMC, Vrije Universiteit, Cancer Center Amsterdam, Amsterdam, The Netherlands; 22 Department of Surgery, Netherlands Cancer Institute, Amsterdam, The Netherlands; 23 Department of Radiology, Amphia Hospital, Breda, The Netherlands; 24 Department of Radiology, Máxima Medical Centre, Veldhoven, The Netherlands; 25 Department of Surgery, Máxima Medical Centre, Veldhoven, The Netherlands; 26 Department of Surgery, Medical Spectrum Twente, Enschede, The Netherlands; 27 Department of Radiology, Groeninge Hospital, Kortrijk, Belgium; 28 Department of Radiology, Netherlands Cancer Institute, Amsterdam, The Netherlands; 29 Department of Surgery, OLVG, Amsterdam, The Netherlands; 30 Department of Radiology, Poliambulanza Foundation Hospital, Brescia, Italy; 31 Department of Radiology, University Medical Centre Groningen, Groningen, The Netherlands; 32 Department of Radiology, OLVG, Amsterdam, The Netherlands; 33 Department of Surgery, St. Antonius Hospital, Nieuwegein, The Netherlands; 34 Department of Surgery, IJsselland Hospital, Capelle aan den IJssel, The Netherlands; 35 Department of Surgery, Hospital Oost-Limburg, Genk, Belgium; 36 Department of Radiology, Erasmus MC, University Medical Center Rotterdam, Rotterdam, The Netherlands; 37 Department of Surgery, Amsterdam UMC, Vrije Universiteit, Cancer Center Amsterdam, Amsterdam, The Netherlands; 38 Department of Epidemiology and Data Science, Amsterdam UMC, University of Amsterdam, Amsterdam, The Netherlands

**Keywords:** Colorectal cancer, Colorectal liver metastases, Liver metastases, Liver MRI, Abdominal CT scan, Diffusion weighted imaging, Gadoxetic acid, Hepatic resection, Liver surgery, Thermal ablation

## Abstract

**Background:**

Abdominal computed tomography (CT) is the standard imaging method for patients with suspected colorectal liver metastases (CRLM) in the diagnostic workup for surgery or thermal ablation. Diffusion-weighted and gadoxetic-acid-enhanced magnetic resonance imaging (MRI) of the liver is increasingly used to improve the detection rate and characterization of liver lesions. MRI is superior in detection and characterization of CRLM as compared to CT. However, it is unknown how MRI actually impacts patient management. The primary aim of the CAMINO study is to evaluate whether MRI has sufficient clinical added value to be routinely added to CT in the staging of CRLM. The secondary objective is to identify subgroups who benefit the most from additional MRI.

**Methods:**

In this international multicentre prospective incremental diagnostic accuracy study, 298 patients with primary or recurrent CRLM scheduled for curative liver resection or thermal ablation based on CT staging will be enrolled from 17 centres across the Netherlands, Belgium, Norway, and Italy. All study participants will undergo CT and diffusion-weighted and gadoxetic-acid enhanced MRI prior to local therapy. The local multidisciplinary team will provide two local therapy plans: first, based on CT-staging and second, based on both CT and MRI. The primary outcome measure is the proportion of clinically significant CRLM (CS-CRLM) detected by MRI not visible on CT. CS-CRLM are defined as liver lesions leading to a change in local therapeutical management. If MRI detects new CRLM in segments which would have been resected in the original operative plan, these are not considered CS-CRLM. It is hypothesized that MRI will lead to the detection of CS-CRLM in ≥10% of patients which is considered the minimal clinically important difference. Furthermore, a prediction model will be developed using multivariable logistic regression modelling to evaluate the predictive value of patient, tumor and procedural variables on finding CS-CRLM on MRI.

**Discussion:**

The CAMINO study will clarify the clinical added value of MRI to CT in patients with CRLM scheduled for local therapy. This study will provide the evidence required for the implementation of additional MRI in the routine work-up of patients with primary and recurrent CRLM for local therapy.

**Trial registration:**

The CAMINO study was registered in the Netherlands National Trial Register under number NL8039 on September 20th 2019.

**Supplementary Information:**

The online version contains supplementary material available at 10.1186/s12885-021-08833-1.

## Background

Colorectal cancer is the third most common cancer and the second most common cause of cancer related death worldwide [1]. Approximately 30% of patients with colorectal cancer present with colorectal liver metastases (CRLM) or will develop CRLM during follow-up [2–4]. Surgery is the cornerstone of curative intent therapy for these patients with 5-year survival exceeding 40% [3, 5, 6]. In recent years, local ablative techniques are increasingly performed to treat small CRLM showing comparable long-term outcomes to surgical resection in these selected patients [7–11]. Both surgery as well as thermal ablation require accurate detection of CRLM. Therefore, optimal pre-interventional localization of CRLM is of utmost importance to determine an adequate local therapy plan.

The current routine diagnostic workup of patients with suspected CRLM consists of contrast-enhanced computed tomography (CT) [12–14]. While CT provides adequate whole-body staging and has a sensitivity of 70–90% for CRLM lesions of more than 10 mm, the reported sensitivity of CT in the detection of small CRLM (≤10 mm) ranges from 22 to 68% [15–17]. This suggests that CT might not be the optimal diagnostic tool to characterize small liver lesions. In recent years, magnetic resonance imaging (MRI) and positron emission tomography CT with fluorine 18 fluorodeoxyglucose (FDG-PET-CT) have been used in addition to CT to increase accuracy [18–20]. Previous studies reported a lower sensitivity (74.1% vs. 82.1%) of FDG-PET-CT and a higher specificity (93.9% vs. 73.5%) as compared to CT [21]. A recent multicentre randomized trial demonstrated that FDG-PET-CT resulted in a modest change in surgical management of only 8.7%, not meeting the predefined minimum incremental diagnostic accuracy of 25% in that study, without an effect on overall survival [22].

Liver MRI is increasingly used as an additional imaging modality for staging of CRLM since it is considered to be more accurate for detection of CRLM as compared to CT [23–26]. To improve the performance of liver MRI, several different contrast agents have been developed, such as Gadoxetic acid (Primovist™, Bayer Schering Pharma, Berlin, Germany). Gadoxetic acid is a liver-specific MR contrast agent that has both dynamic and hepatocyte-specific properties, improving detection and characterization of focal liver lesions [27]. For the detection of CRLM, a gadolinium contrast-enhanced MRI scan has a sensitivity and specificity of 90 and 87%, respectively, while the reported sensitivity and specificity of gadoxetic acid contrast-enhanced MRI is 87–100 and 95%, respectively [24, 28]. Furthermore, MRI with diffusion-weighted imaging (DWI) in addition to gadoxetic acid enhanced MRI may substantially increase the accuracy of detection of focal liver lesions and reduce overdiagnosis of CRLM [29–31].

Although previous literature suggests that liver MRI is superior in detection and characterization of CRLM compared to CT, it is not known whether the higher diagnostic accuracy of MRI has sufficient impact on patient management in terms of additional clinically significant CRLM (CS-CRLM) that actually change the local therapy plan. The current European Society for Medical Oncology (ESMO) consensus guideline for the management of patients with metastatic colorectal cancer recommends the use of MRI in the pre-interventional workup of CRLM, but indicates that more evidence is needed to address the clinical added value of MRI in patients with CRLM [14]. In addition, the American College of Radiology (ACR) Appropriateness Criteria—Pretreatment Staging of Colorectal Cancer highlighted the difficulty to determine the best imaging modality for patients with CRLM because very few studies have adequately compared the accuracy of MRI to high-quality CT [13]. Since the available evidence supports that both MRI and CT detect liver lesions with high accuracy, the ACR Appropriateness Criteria state that CT or MRI may be used to stage CRLM [13]. These inconclusive guideline recommendations stress the lack of robust data and thorough scientific evidence in this field. Hence, the actual role of liver MRI for management of patients with CRLM remains unclear. Until now, no definitive evidence has been provided on the impact of liver MRI on finding CS-CRLM as compared to CT for patients with primary or recurrent CRLM.

The aim of the present CAMINO study is to evaluate whether diffusion-weighted and gadoxetic acid enhanced liver MRI has sufficient clinical added value to CT in order to be routinely performed in the staging of CRLM. This is an international multicentre prospective diagnostic accuracy study assessing the proportion of patients with CRLM scheduled for local therapy in which liver MRI finds additional CS-CRLM as compared to CT. The hypothesis is that the higher accuracy of liver MRI will lead to finding of at least 10% additional CS-CRLM compared to CT, resulting in improved decision-making on the local therapy plan, such as additional surgery or ablation and potentially preventing unnecessary surgical exploration or ablative treatment. The secondary aim of this study is to identify subgroups which benefit most from additional liver MRI.

## Design/methods

### Study design

The CAMINO study is an international multicentre prospective incremental diagnostic accuracy study on the added value of liver MRI including DWI and gadoxetic acid enhanced T1-weighted sequence in patients with CRLM who are considered candidates for local therapy based on CT. Patients will be recruited in 17 liver surgery centres in the Netherlands, Belgium, Norway and Italy: Amsterdam UMC, Amsterdam; Erasmus University Medical Center, Rotterdam; Leiden University Medical Center, Leiden; University Medical Center Groningen, Groningen; Netherlands Cancer Institute, Amsterdam; OLVG, Amsterdam; Amphia Hospital, Breda; Medical Spectrum Twente, Enschede; Jeroen Bosch Hospital, ‘s Hertogenbosch; St. Antonius Hospital, Nieuwegein; Maxima Medical Center, Veldhoven; IJsselland Hospital, Capelle aan den IJssel; Albert Schweitzer Hospital, Dordrecht (all in the Netherlands); Oslo University Hospital, Oslo, Norway; AZ Groeninge, Kortrijk, Belgium; Hospital Oost-Limburg, Genk, Belgium and Poliambulanza Foundation Hospital, Brescia, Italy. Due to the large number of participating centres, a phased implementation of the start of the study will be used.

All participating centres have a formal multidisciplinary team (MDT) meeting at least once per week for patients with CRLM and all have extensive experience in the diagnostic workup and in both liver surgery and thermal ablation, defined as performing ≥20 procedures annually. Treating surgeons and interventional radiologists are board certified and have performed and/or supervised at least 100 procedures.

The study is formally endorsed by the Dutch Colorectal Cancer Group (DCCG), the Abdominal Radiology section of the Radiological Society of the Netherlands (NVvR) and the Dutch Colorectal Patient Foundation (Stichting Darmkanker). The study is funded by the Dutch Cancer Foundation (KWF Kankerbestrijding) and co-funded by Bayer Schering Pharma® (Berlin, Germany) in terms of providing Gadoxetic acid for all patients. The Dutch Cancer Foundation and Bayer Schering Pharma® (Berlin, Germany) had no role in the design of the study and will not have any influence on the collection of data, data analysis, interpretation of data, writing of manuscript, and decision to publish. The study is registered in the Netherlands National Trial Register (NL8039, September 20th 2019).

This study will be conducted in accordance with the guidelines of Good Clinical Practice and in agreement with the Declaration of Helsinki (64th version, October 2013). The Medical Ethical Review Board (METc) of the Amsterdam UMC, location AMC, has assessed primarily that the CAMINO study was NOT subject to the Medical Research Involving Human Subjects Act (WMO). The METc of every participating hospital supported the decision of the METc of the Amsterdam UMC, location AMC and approved their participation in this study by signing a non-interventional study agreement. For designing this study protocol, the SPIRIT 2013 and STARD 2015 recommendations were used [32, 33] (SPIRIT Checklist provided in Additional file 1).

### Objectives and hypotheses

The main objective of this study in patients with CRLM is to identify the proportion of patients in which liver MRI including DWI and gadoxetic acid enhanced T1-weighted sequence finds CS-CRLM in addition to an abdominal portal venous phase CT. CS-CRLM are defined as liver lesions that have not been incorporated in the initial resection and/or ablation plan based on CT, hence leading to a change in local therapy. Change in local therapy is defined as any change in surgery or thermal ablation including increase or decrease of the number or extent of liver resections, increase or decrease in number of ablation zones, surgery to ablation, ablation to surgery, addition of ablation to surgery and cancellation of local therapy. The local therapy plan proposed by the MDT based on CT only will be compared to the local therapy plan based on CT and liver MRI. It is hypothesized that liver MRI will lead to the detection of CS-CRLM in at least 10% of patients which is considered the minimal clinically important difference. The findings of this study – whether or not liver MRI including DWI and gadoxetic acid enhanced T1-weighted sequence has clinical added value on local therapy strategy of CRLM patients – will determine the role of liver MRI in the staging of CRLM and will provide international guidelines with data for a scientifically based recommendation on the implementation of liver MRI in the pre-interventional workup.

The secondary aim is to identify which subgroups benefit most from the additional liver MRI by performing a logistic regression analysis and developing a clinical prediction model.

### Study population

Consecutive adult patients with primary or recurrent CRLM who are considered candidates for curative surgery, thermal ablation or a combination of curative surgery and intra-operative ablation, based on abdominal CT will be invited to the study. Patients who receive neoadjuvant or induction chemotherapy and are subsequently eligible for local curative therapy will be invited as well.

#### Inclusion criteria

To be included, patients must fulfil all of the following criteria:

- Histological proof of primary colorectal cancer
- Age ≥ 18 years.
- Patients with primary or recurrent CRLM who are considered candidates for curative surgery, thermal ablation or a combination of curative surgery and intra-operative thermal ablation, determined during MDT based on abdominal CT.
- Written informed consent.

#### Exclusion criteria

Patients fulfilling one or more of the following criteria will be excluded:

- Contraindications for liver surgery or thermal ablation.
- Extrahepatic metastases on CT-chest/abdomen and no realistic option for curative therapy.
- Unresectable primary tumor, or resectable primary tumor requiring immediate surgery.
- Patients with contra-indications for liver MRI.
- Previous inclusion in the CAMINO study.

### Informed consent

Informed consent will be obtained for the use of individual patient data for study purposes. The formal written informed consent will be obtained during the outpatient clinic visit by a member of the study team. By signing the informed consent form, patients agree to participate in this study. Before patients agree to participate in this study, they will be provided with written information in the form of a Patient Information Sheet. All patients will be informed about the strict confidentiality of their patient data, and that their medical records may be reviewed for study purposes by authorized individuals other than their treating physician. Participants will be told that they may withdraw from the study at any time for any reason. This will not have any influence on subsequent care. Patients who do not want to participate in the study will have the current routine preoperative workup. Patients withdrawing permission will be replaced.

Liver MRI is a non-invasive imaging method used in daily clinical practice and under intensive quality control as part of its clinical applications. Safety reporting and a data safety monitory board (DSMB) are therefore not implemented in this study as there are no added risks.

### Study procedures

#### Preoperative imaging

In this study, all participants will undergo both abdominal CT and liver MRI. An abdominal CT will include a contrast-enhanced CT scan using a portal venous phase protocol. Liver MRI includes at least T2-weighted sequence, T1-weighted sequence with gadoxetic acid as contrast agent (i.e. plain, arterial, portal venous, early delayed and late delayed phase) as previous studies showed that gadoxetic acid has become the preferred contrast agent in liver MRI for CRLM, and DWI [24, 30]. Since gadoxetic acid will be administered immediately before the start of MRI, the duration of the scan will not exceed the standard duration with any other contrast agent. In every participating centre there is substantial experience with the use of gadoxetic acid and also with interpretation and assessment of gadoxetic acid enhanced liver MRI.

Both CT and liver MRI will be performed according to the imaging protocols from the Radiological Society of the Netherlands (Additional file 2). These protocols give guidance and are considered as minimal requirements for scanning in all participating centres. Prior to the start of the study, the protocol per scanner is retrieved from each study site and checked to see whether it meets the minimum requirements concerning type of scanner, dose of iodinated contrast, scan delay, kilovoltage dose, collimation and slice thickness for CT and magnetic field strength, type of contrast agent, dose of contrast agent, scan series, scan sequences and b values for DWI for MRI. Patients referred from other centres to the participating center with prior CT (and MRI) are also eligible. Imaging from those referral centres can be used, but only when it meets the protocol requirements of the Radiological Society of the Netherlands. If the abdominal radiologist from the participating center deems that imaging quality does not meet protocol requirements, imaging has to be repeated.

All pre-interventional imaging will be assessed by an experienced abdominal radiologist. Liver MRI will be assessed by a radiologist who is not blinded to CT results since liver MRI is performed in addition to CT in daily clinical practice. The radiologist has access to information about previous CT results and the interpretation of the liver MRI may be influenced by this additional information. However, we aim to evaluate the clinical added value of liver MRI in addition to CT and this reflects how both examinations are used in daily clinical practice.

The aim is to perform all preoperative examinations within a window of 6 weeks prior to the day of surgery and/or ablation, with an extension possible to a maximum of 10 weeks. The time interval between CT and liver MRI is 4 weeks at maximum. This time interval for CT and MRI is based on the rapid growth potential of CRLM and the importance of CT and MRI being performed within a sufficiently short time interval while taking into account the feasibility of arranging liver MRI in participating centres.

If there are contraindications for gadoxetic acid, liver MRI is performed with another routinely used extracellular contrast medium at the participating center or –when contraindicated– no contrast medium is used. A small subset of patients may undergo FDG-PET-CT instead of CT. These patients are also eligible to participate. However, we will perform a subgroup analysis excluding patients that underwent FDG-PET-CT.

#### Multidisciplinary team meeting

**Fig. 1:**
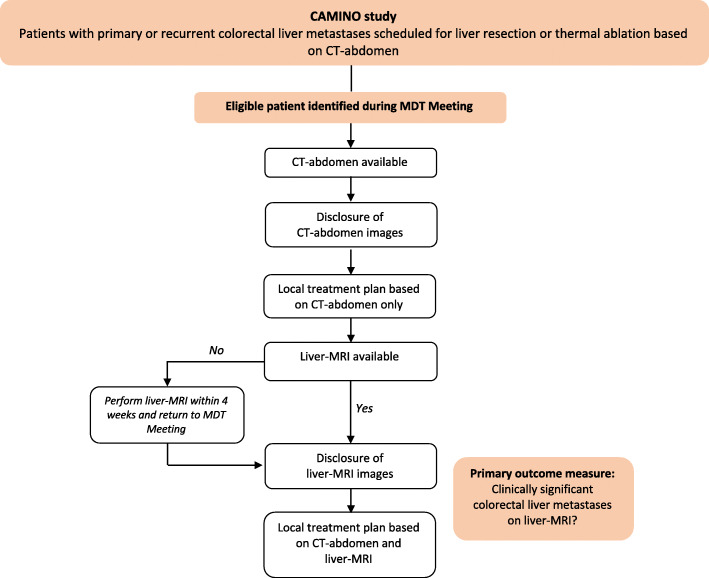
Flowchart of study procedure during multidisciplinary team meeting

In all participating centres, potentially eligible patients will be discussed during a formal regular MDT meeting (Fig. 1). First, the CT will be presented by the radiologist and indication for local therapy will be determined by the MDT. If a patient is considered candidate for local therapy, the exact surgical or ablative plan based on CT only will be decided and documented in the electronic case report form (eCRF). After finalizing and documenting the CT-based local therapy plan, the findings of liver MRI are disclosed as a second step. This will be either – if directly available – at the same MDT meeting, or at the next MDT meeting, after liver MRI has been performed (no longer than 4 weeks after CT). CS-CRLM found at liver MRI with the exact change in the local therapy plan will be documented in the eCRF. CS-CRLM are defined as liver metastases that have not been incorporated in the initially planned resection or ablation based on CT, therefore leading to a change in local therapeutical management. Change in local therapy plan is defined as any change in surgery or thermal ablation including increase or decrease of the number or extent of liver resections, increase or decrease in number of ablation zones, surgery to ablation, ablation to surgery, addition of ablation and cancellation of local therapy.

#### Intra-operative imaging and histological findings

During surgery, intraoperative ultrasound (IOUS) of the liver is performed by the surgeon or the radiologist followed by the actual surgical or ablative treatment. IOUS is not part of the primary aim of this study and is performed especially to determine surgical margins and identification of vascular structures and not primarily for lesion detection and characterization. We will register whether IOUS has found new CS-CRLM in addition to the preoperative CT and liver MRI, and whether the findings of the IOUS changed the surgical or ablative plan. Additionally, we will strive to report whether postoperative histopathology reports were in accordance with findings of the pre-operative CT, liver MRI and IOUS.

### Follow-up

All patients who underwent surgical and/or ablative treatment will be assessed in the local follow-up schedule of the study site. Ideally, patients will be seen with a CT-chest/abdomen at 3 to 4 months and 6 to 8 months after surgery and/or thermal ablation. Patients who are no longer considered as candidates for local curative therapy based on the additional liver MRI findings will have at least 6 months follow-up by CT, as stated in ESMO guidelines^13^. Findings regarding disease recurrence or new CRLM, possible treatment options and overall patient survival will be documented during at least 6 months follow-up.

### Expert panel procedure and evaluation

In addition to the primary outcome measure which will be determined in the daily clinical workflow of the participating centres, a fixed central independent, blinded expert panel is created to validate the findings of the individual centres as a secondary outcome measure. The expert panel consists of three abdominal radiologists, two interventional radiologists and three liver surgeons. These are recruited from participating centres and will post-hoc evaluate step-by-step the additional value of liver MRI as compared to CT only on the local therapy plan. The participating radiologists have extensive experience with CT and liver MRI and have assessed at least 500 abdominal CT scans and 500 liver MRI scans for focal liver lesions. The participating liver surgeons performed at least 100 liver resections. The participating interventional radiologists have extensive experience in performing thermal ablation of CRLM and performed at least 100 liver ablation procedures. The CT and liver MRI of each patient will be assessed by one radiologist only since all radiologists have extensive experience in assessing abdominal CT scans for focal liver lesions and liver MRI scans. Radiologists will not review examinations from their own centre. Based on CT and liver MRI findings of the radiologist, each interventional radiologist and surgeon will get the findings presented and will indicate independently whether they would change their initial local therapeutical plan based on the liver MRI results. Agreement of 4/5 expert panel members (agreement rate of 80% or more) will be considered as consensus. In case of agreement of three or less expert panel members, a joint consensus meeting will be organized to discuss the respective cases and eventually reach consensus.

### Study outcomes

The primary outcome of the current study is the proportion of patients with a change in local therapy plan due to CS-CRLM on liver MRI, not seen on CT. CS-CRLM are defined as liver lesions that have not been incorporated in the initial resection and/or ablation plan based on CT, hence leading to a change in local therapy. Change in local therapy is defined as any change in surgery or thermal ablation including increase or decrease of the number or extent of liver resections, increase or decrease in number of ablation zones, surgery to ablation, ablation to surgery, addition of ablation to surgery and cancellation of local therapy. The local therapy plan proposed by the MDT based on CT only will be compared to the local therapy plan based on CT and liver MRI.

Secondary outcomes include a prediction model to evaluate the probability of finding CS-CRLM on liver MRI and the proportion of patients with a change in local therapy plan based on the occurrence of CS-CRLM on liver MRI, as identified by the expert panel in the post hoc evaluation.

### Data collection

All parameters will be collected prospectively. Relevant patient data (e.g. patient characteristics, medical history, primary tumor information, local or recurrent CRLM, neoadjuvant chemotherapy, laboratory results, pathology report and follow-up data) will be retrieved from the electronic patient record. Imaging results will be collected according to a standardized radiology report, including the following parameters: number, size and localization of lesions, vascular involvement of lesion (i.e. involvement of portal and/or hepatic veins), characterization of lesions (i.e. indeterminant or definitely malignant). For MRI, the sequence on which the lesion was seen best will also be documented. Postoperative data will be obtained for 30 days, including complications, length of hospital stay, readmission and mortality. Participating centres are asked to collect and share their images of the pre-interventional CT chest/abdomen and liver MRI in order to be assessed by the expert panel. All patient data and imaging will be coded by an individual study number and saved in the eCRF. No identifying data will be entered into the database. All data and patient imaging will be stored in an online database specially designed to share patient imaging in a safe and privacy-respecting environment (ALEA®, FormsVision, Abcoude, the Netherlands). Safety reporting and a DSMB are not implemented in this study as there are no added risks. This study will follow the FAIR principles in handling and storage of data [34].

### Statistical analysis plan

Since the CAMINO study has a pragmatic design and should represent daily clinical practice, the main analysis of the primary outcome will be based on the intention-to-imaging (ITI) population. The ITI population will consist of all included patients fulfilling the inclusion criteria of the CAMINO study, regardless of any study protocol violation such as an extended time interval of more than 4 weeks between CT and MRI and liver MRI performed with another contrast agent then Gadoxetic acid. The incremental accuracy in the ITI analysis will be calculated as the number of included participants with a clinically significant CRLM (CS-CRLM) detected by liver MRI and not seen on CT, as identified by the local MDT, relative to the total number of included participants.

Additionally, the main analysis will be complemented by an imaging-per-protocol (IPP) analysis. The IPP population will include all patients fulfilling the inclusion criteria and undergoing all study procedures per study protocol, excluding patients who showed any study protocol violation or deviation. The incremental accuracy in the IPP analysis will be calculated as the number of included participants with a clinically significant CRLM (CS-CRLM) detected by liver MRI and not seen on CT, relative to the total number of included participants without .

Three subgroup analyses will be performed: first, in the groups of patients who did and did not receive preoperative chemotherapy; second, in patients who did and did not undergo a FDG-PET-CT, and at last, based on the number of CRLM.

A prediction model will be developed using multivariable relaxed lasso logistic regression modelling, to evaluate the probability based on patient, tumor and procedural variables of finding CS-CRLM on liver MRI. This exploratory model will be used for identifying subgroups in whom the probability of finding CS-CRLM is less than 10%. All baseline variables will be considered for the regression model.

All baseline, clinicopathological and procedural variables will be described and analysed. Continuous, not normally distributed variables will be expressed as median with interquartile range (IQR). In case variables are normally distributed they will be reported as mean with standard deviation (SD). A Mann-Whitney U test will be used to compare continuous, not normally distributed variables between groups. Normally distributed, continuous variables will be compared using an Independent samples T-test. Categorical variables will be reported as frequencies and proportions and compared between groups using a chi-square test. *P*-values below 0.05 will be considered significant. Data analysis will be performed using IBM SPSS Statistics for Windows© version 25.0 (SPSS Inc., Chicago, IL, USA).

### Sample size calculation and statistical considerations

The literature on the clinical impact of additional liver MRI in patients with primary or recurrent CRLM is very limited and consists of primarily small retrospective studies [26, 35]. Our estimate of the chances of finding CS-CRLM on liver MRI is based on literature, clinical experience and practicality.

A smaller change in local therapy plan is expected than previous studies have shown, as CT technique and interpretation have evolved. Based on these considerations, the proportion of patients in which liver MRI finds CS-CRLM is postulated to be 10%. Such a proportion can be considered as a relevant impact on local therapeutical management.

The primary outcome measure, the proportion of patients with CS-CRLM on liver MRI not identified on CT, guides the sample size for this incremental diagnostic accuracy study. Taking into account that all patients will undergo CT and liver MRI, precision is defined as one-sided 95% confidence interval (CI) with a lower confidence bound of 6.5%. Sample size calculation indicated that 270 patients need to be included in order to obtain estimates of change in management in 10% with 95% CIs that does not exceed 7%.

A small subset of the patients will undergo FDG-PET-CT instead of CT. During a recent national meeting of the Dutch Liver Collaborative Group, an assessment about the use of FDG-PET-CT was performed and approximately 5% of patients in this study is expected to undergo FDG-PET-CT instead of CT. To adjust for patients that undergo FDG-PET-CT instead of CT, an increase of 5% of the desired number of participants is made, resulting in 284 patients. Given the design of the study, a maximum dropout of 5% is expected. Taken this dropout into account, a total of 298 patients must be enrolled in this study. If a participant withdraws consent, a new one will be recruited.

## Discussion

It is unknown to what extent liver MRI may result in a change in clinical management in patients with CRLM scheduled for local therapy based on staging with CT. Current evidence is based on single centre retrospective studies. The present international multicentre prospective study will determine the clinical added value of liver MRI to CT in patients with CRLM scheduled for local curative therapy including liver resection and/or thermal ablation.

A non-randomized design was chosen since a randomized controlled trial comparing abdominal CT and liver MRI versus CT was considered not feasible due to the high use of MRI in current daily practice. A retrospective analysis to document the incremental value of liver MRI compared to CT is not reliable, since a regular MDT meeting structure does not include a consistent step-by-step documentation of the added value of MRI on final patient management.

In contrast, it is also important to acknowledge the burden of performing an additional liver MRI in the workup of patients with CRLM. Performing routine liver MRI in all CRLM patients may add diagnostic delay due to waiting lists and limited MRI-capacity. Indeterminate lesions at liver MRI could make it more difficult to decide on an accurate surgical or ablative strategy. In addition, CT in combination with surgical exploration and IOUS is considered as the standard for CRLM detection and is performed in all patients, questioning the added value of liver MRI and its associated costs. As adding routine liver MRI to the workup of CRLM patients may cause additional pre-interventional delays and increases healthcare costs, any recommendation about its use should be guided by high quality studies which determine its exact added value.

With the participation of 17 liver centres in four European countries, a broad spectrum of patients will be included and the current perioperative care of patients with CRLM will be outlined. Assessment of imaging and decisions on local therapeutical management will be performed by dedicated abdominal and interventional radiologists and hepatobiliary surgeons, which may introduce variation. Through the participation of a large number of liver centres, the data collected in this pragmatic diagnostic accuracy study will reflect daily clinical practice, which will add to the generalizability of our findings. The use of an additional post-hoc expert panel will elaborate the meaning of this possible interobserver variation and will give insight into findings in the prospective cohort [36]. We will strive to determine the final histopathology of the detected CS-CRLM, although it is acknowledged that at times this may be difficult, especially in the scenario of a large number of CRLM.

In summary, this prospective international multicentre study will provide the required evidence to decide on the routine use of liver MRI during the preoperative workup of patients with primary and recurrent CRLM considered candidates for local therapy. Furthermore, this study will attempt to determine which patients benefit most from such an approach, and potentially which patients do not.

## Supplementary Information

**Additional file 1.** SPIRIT checklist

**Additional file 2.** Imaging Protocols of The Radiological Society of the Netherlands

## Data Availability

The datasets generated and/or analyzed during the current study are not publicly accessible but are available from the corresponding author on reasonable request, as well as the full study protocol.

## Abbreviations

CRLM: Colorectal liver metastases
CT: Computed Tomography
MRI: Magnetic Resonance Imaging
FDG-PET-CT: Positron Emission Tomography CT with fluorine 18 fluorodeoxyglucose
DWI: Diffusion-weighted imaging
CS-CRLM: Clinically significant colorectal liver metastases
MDT: Multidisciplinary team
METc: Medical Ethical Review Board
eCRF: Electronic case report form
IOUS: Intraoperative ultrasound
IQR: Interquartile range
SD: Standard deviation
ITI: Intention-to-imaging
IPI: Imaging-per-protocol
CI: Confidence interval
