# Supplementary material for: Clinical added value of MRI to CT in patients scheduled for local therapy of colorectal liver metastases (CAMINO): study protocol for an international multicentre prospective diagnostic accuracy study
Source: BMC Cancer. 2021 Oct 18;21:1116. doi: 10.1186/s12885-021-08833-1 (PMC8524830; doi:10.1186/s12885-021-08833-1)
Supplement: Supplementary file 2 — Additional file 2. Imaging Protocols of The Radiological Society of the Netherlands [file 12885_2021_8833_MOESM2_ESM.docx]

**Additional File 2. Imaging Protocols of The Radiological Society of the Netherlands**

**1. Abdominal CT**

This study scan proposal is based on the abdominal CT scan protocol of The Radiological Society of the Netherlands. This protocol gives guidance and should be considered as minimal requirements.

| Preparation | |
| --- | --- |
| Oral contrast medium | Iodinated contrast medium, water or no oral contrast medium |

| Series | |
| --- | --- |
| 1 Scanner | At least 64-slice MDCT |
| 2. Chest/abdomen | Include complete lungs to groins |
| 3. Patient position | Supine, arms up |
| 4. Instructions | Inspiration |
| 5. Iv contrast | 70-150 ml iodinated contrast medium (concentration 300 mg I/ml), saline flush |
| 6. Scan | Superior-inferior |
| 7. Delay | Scan delay ≈ 65 s -70s or bolus tracking |
| 8. KV | 100 |
| 9. Collimation, slice thickness | Smallest collimation  64-slice: 0.5-0.625 mm, pitch 0.9-1.1  >64-slice: 0.5-0.625 mm, pitch 0.7-0.9 |

**2. Liver-MRI**

This study scan proposal is based on liver-MRI scan protocol of The Radiological Society of the Netherlands. This protocol gives guidance and should be considered as minimal requirements.

| **Preparation** | |
| --- | --- |
| Oral contrast | None |

| Series | | |
| --- | --- | --- |
| 1.MRI | 1.5 or 3.0 T |  |
| 2. Patient position | Feet first, Supine |  |
| 3. Coil | Dedicated abdominal coil |  |
| 4. Iv contrast | Primovist, 0.1 ml/kg |  |
| 5. Series  (minimal requirements) | - T2 coronal  - T2 axial  - DWI axial, b= 0-1000 (at least two b values, at least one above 600)  - Axial T1 FS dynamic series with Primovist:  Plain (t=0), arterial (t=30 sec), portovenous (t=70sec), early delayed (t= 180 sec) and late  delayed phase (t=10 min; in cirrhotic patients t=20 min) | *If Primovist is contraindicated, MRI is performed with an extracellular contrast agent or without any contrast agent,* |
| 6. Slice thickness | Maximum 5mm, for T1 maximum 3 mm |  |
